# Marine Natural Products from the Yucatan Peninsula

**DOI:** 10.3390/md18010059

**Published:** 2020-01-16

**Authors:** Dawrin Pech-Puch, Mar Pérez-Povedano, Oscar A. Lenis-Rojas, Jaime Rodríguez, Carlos Jiménez

**Affiliations:** Centro de Investigacións Científicas Avanzadas (CICA) e Departmento de Química, Facultade de Ciencias, Universidade da Coruña, 15071 A Coruña, Spain; dawrin.j.pech@udc.es (D.P.-P.); perezpovedanomaranabel@gmail.com (M.P.-P.); oscar.lrojas@udc.es (O.A.L.-R.)

**Keywords:** natural products, Yucatan peninsula, marine biodiversity

## Abstract

Mexico is one of the three areas of the world with the greatest terrestrial and cultural biological diversity. The diversity of Mexican medicinal flora has been studied for a long time and several bioactive compounds have been isolated. The investigation of marine resources, and particularly the potential of Mexican marine resources, has not been intensively investigated, even though the Yucatan Peninsula occupies 17.4% of the total of the Mexican coast, with great biological diversity in its coasts and the ocean. There are very few studies on the chemistry of natural products from marine organisms that were collected along the coasts of the Yucatan Peninsula and most of them are limited to the evaluation of the biological activity of their organic extracts. The investigations carried out on marine species from the Yucatan Peninsula resulted in the identification of a wide structural variety of natural products that include polyketides, terpenoids, nitrogen compounds, and biopolymers with cytotoxic, antibacterial, antifouling, and neurotoxic activities. This review describes the literature of bioprospecting and the exploration of the natural product diversity of marine organisms from the coasts of the Yucatan Peninsula up to mid-2019.

## 1. Introduction

The potential of marine natural products in drug discovery is invaluable due to the extremely rich biodiversity of the marine environment. The marine environment contains a large number of species which are the source of a wide range of structurally diverse bioactive secondary metabolites. Approximately 29,000 marine natural products are known, from which eight compounds have become commercialized drugs. During the last decade, more than 1000 new marine natural products have been annually isolated, but the set of new and unique structures is far from being exhausted [1,2]. The interest in marine organisms has been increasing, since they are capable of producing a great diversity of novel metabolites, such as unusual nucleosides, bioactive terpenes, sterols, cyclic peptides, alkaloids, fatty acids, peroxides, and amino acid derivatives, many of them with high potential for pharmacological applications [3].

The Yucatan Peninsula in Mexico, which comprises the Mexican states of Campeche, Quintana Roo, and Yucatan, is known as a biotic province [4]. Although marine organisms constitute a recognized source of a wide range of structurally diverse natural products, research that is focused on marine natural products from the Yucatan Peninsula is still in infancy, mainly when compared to the numerous studies of those isolated from terrestrial organisms, especially from plants [5].

On the other hand, a priority task for the conservation and management of coastal areas, such as the Yucatan Peninsula, and for the discovery of new sources of novel natural products, is the study of their biodiversity. The first effort aimed at determining the state of health of the coast of the Yucatan Peninsula was reported in 2010. Pech-Pool and Ardisson Herrera described the identification of more than 400 thousand organisms from marine and coastal environments and lagoons, belonging to 529 species, which were distributed in 13 phyla, 26 classes, 28 orders, 113 families, and 358 genera. Of the registered species, 45% (237) corresponded to the Arthropod (Crustacea); 22% (118) to Mollusca; 14% (72) to the Nematoda; and, 13% (68) to the Annelida phyla. The remaining 6% (33) belonged to the Echinodermata, Nemertea, Platyhelminthes, Sipuncula, Porifera, Chaetognatha, Chordata, and Cnidaria phyla [6]. More specifically, taxonomic identification of coral and sponges was also described. Thus, from a total of 31 registered coral species, 15 corresponded to order Scleractinia, being Poritidae and Faviidae the most important families that were represented by four species each; other 15 species were octocorals, seven of them belonged to Plexauridae family and, finally, the remaining species was a hydrocoral [7]. On the other hand, most of the registered sponges belonged to three classes: Calcarea, Hexactinellida and Demospongiae, the last being the most predominant and with the greatest diversity. The 50 species registered were distributed in 10 orders, two subclasses, 25 families, and 35 genera [8]. The results of studies that were focused on marine biodiversity in specific benthic communities were also reported. For example, the biodiversity analysis of the Alacranes Reef, one of the largest platform-type reefs in Mexico, covering an approximate area of 333.7 km^2^, showed that this benthic community mainly consists of macroalgae (50.1%), seagrass (16.2%), algal mat (13.6%), scleractinia corals (11.1%), octocorals (7.6%), sponges (0.6%), and other vagile and sessile organisms (0.5%) and hydrocorals (0.3%) [9]. A very recent report published in 2019 describes 31 ascidian species from the Yucatan Peninsula that were grouped into 13 families and 19 genera, being two species, *Clavelina* sp. and *Pyura* sp., described for the first time [10].

With the present review, we will cover the current knowledge of bioprospecting and the exploration of the natural product diversity of marine organisms that were collected along the coasts of the Yucatan Peninsula up to mid-2019.

## 2. Marine Natural Products from the Yucatan Peninsula

Although the number of secondary metabolites that were isolated from marine organisms collected along the coasts of the Yucatan Peninsula is not very high, they display a great diversity of structures and biological activities. They can be grouped into the following categories as polyketides (aliphatic polyketides, glycolipids, and aromatic acids), terpenoids (diterpenes and sesterterpenes, steroids, and triterpenoids saponins), nitrogen compounds (indole derivatives, nucleosides, nitrogenous bases, and conotoxins), and biopolymers, based upon the putative biogenetic origins.

The following sections show a detailed list of the isolated natural products from the reported species, along with their biological activities, as well as the taxonomic identification of marine organisms from which they were obtained.

### 2.1. Polyketides

#### 2.1.1. Aliphatic Polyketides

**Figure 1:**
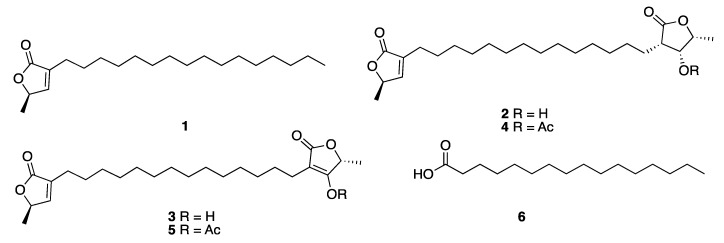
Structures of the aliphatic polyketides **1**–**3**, along with their synthetic acetate derivatives **4** and **5**, isolated from the gorgonian *Pterogorgia anceps* and palmitic acid (**6**) isolated from the sponge *Haliclona tubifera* (now *H.* (*Reniera*) *tubifera*).

The interest on the study of fatty acids derivatives from marine organisms, specifically ω-3 polyunsaturated fatty acids, was sparked by the approval of Lovaza^®^ by the FDA as a mixture of ethyl esters of eicosapentaenoic acid and docosahexaenoic acid used as therapeutic agent for reducing serum triglycerides [1]. The Italian researcher group led by Cimino from Naples published in 1999 the isolation and structure elucidation of three new fatty acids derivatives, the butenolide lipids **1**–**3** from the gorgonian *Pterogorgia anceps*, which were collected at Puerto Morelos, Quintana Roo state. The new fatty acids derivatives were identified as (*R*)-3-hexadecyl-5-methylfuran-2 (5*H*)-one (**1**), (*R*)-3-(14-((3*S*,4*R*,5*R*)-4-hydroxy-5-methyl-2-oxotetrahydrofuran-3-yl)tetradecyl)-5-methylfuran-2(5*H*)-one (**2**), and (*R*)-4-hydroxy-5-methyl-3-(14-((*R*)-5-methyl-2-oxo-2,5-dihydrofuran-3-yl)tetradecyl)furan-2(5*H*)-one (**3**). The proposed stereochemistry was confirmed by acetylation of **2** and **3** to give the acetate derivatives **4** and **5**, respectively [11]. Palmitic acid (**6**) was also isolated from the sponge *Haliclona tubifera* (now *H.* (*Reniera*) *tubifera*) that was collected on the coasts of the Yucatan state [12] (Figure 1).

#### 2.1.2. Glycolipids

**Figure 2:**
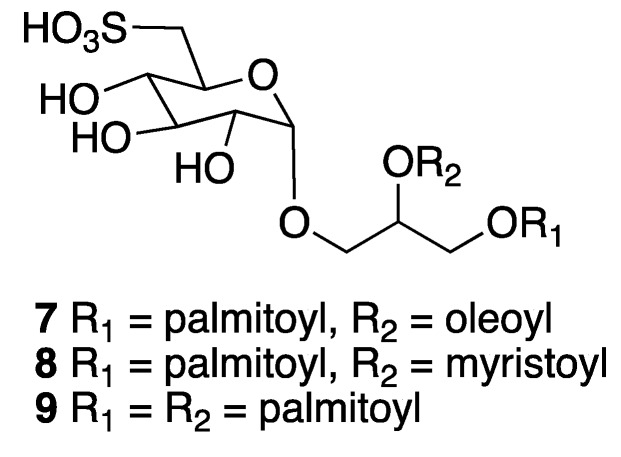
Structures of marine glycolipids isolated from the brown algae *Lobophora variegata*.

Marine glycolipids are amphiphilic compounds that are divided into two main groups: glycoglycerolipids (GGLs) and glycosphingolipids (GSLs). Glycoglycerolipids are composed by a glycerol unit glycosylated at one primary alcoholic function. Sulfoquinovosyldiacylglycerols constitute one of the most common types of glycoglycerolipids (GGLs) found in marine organisms and some of them are present in large amounts in photosynthetic membranes of cyanobacteria, algae and higher plants. Antiviral activity against HIV-1 was reported for some sulfoquinovosyldiacylglycerols that were isolated from cyanobacteria [13]. Freile–Pelegrin and collaborators reported in 2010, from the brown algae *Lobophora variegata*, collected at Puerto Morelos in Quintana Roo state, the isolation, and structure elucidation of a new glycoglycerolipid, 1-*O*-palmitoyl-2-*O*-oleoyl-3-*O*-(6′′′-sulfo-α-d-quinovopyranosyl)-glycerol (**7**), along with two known glycolipids: 1-*O*-palmitoyl-2-*O*-myristoyl-3-*O*-(6′′′-sulfo-α-d-quinovopyranosyl)-glycerol (**8**), and 1,2-di-*O*-palmitoyl-3-*O*-(6′′′-sulfo-α-D-quinovopyranosyl)-glycerol (**9**). The mixture of the three sulfoquinovosyldiacylglicerols **7**–**9** showed high in vitro antiprotozoal activity against *Entamoeba histolytica* (IC_50_ value of 3.9 μg mL^−1^) and moderate activity against *Trichomonas vaginalis* trophozoites (IC_50_ value 8.0 μg mL^−1^), with good selective index (SI > 10). However, they were less effective than metronidazole being used as control (IC_50_ = 0.13 and 0.04 μg mL^−1^/0.759 and 0.230 nM, respectively) [14]. The relative configuration of the glycerol unit in **7**–**9** was not specified (Figure 2).

#### 2.1.3. Aromatic Acids

**Figure 3:**
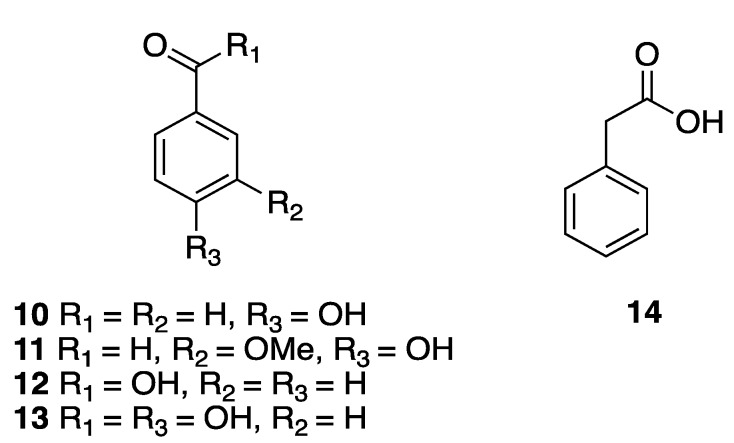
Structures of aromatic acids isolated from the sponge *Haliclona tubifera* (now *H.* (*Reniera*) *tubifera*).

Several aromatic acids, such as *p*-hydroxybenzaldehyde (**10**), vanillin (**11**), benzoic acid (**12**), *p*-hydroxybenzoic acid (**13**), and phenylacetic acid (**14**), were isolated from the sponge *Haliclona tubifera* (now *H.* (*Reniera*) *tubifera*) [12] (Figure 3).

### 2.2. Terpenoids

#### 2.2.1. Diterpenes and Sesterterpenes

**Figure 4:**
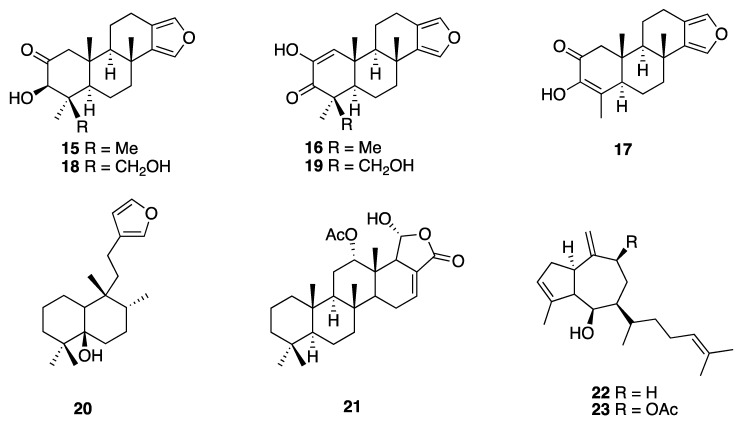
Structures of diterpenes **15**–**20** and sesterterpene **21** isolated from the sponge *Spongia tubulifera* (now *S.* (*Spongia*) *tubulifera*) and diterpenes **22** and **23** isolated from the algae *Dictyota ciliolata.*

Terpenoid biogenesis is one of the dominant pathways of most marine natural products, mainly those that were isolated from cnidarians followed by sponges. The wide variety of biological activities that were found in marine terpenes, together with their ecological role in the marine environment, makes them a very interesting target of study apart from being potential drugs [15]. Pech-Puch et al. reported in 2019 the isolation and structural characterization of seven terpenoids from the sponge *Spongia tubulifera* (now *S.* (*Spongia*) *tubulifera*) that were collected at Rio Indio, Quintana Roo state. Two of them resulted in being new natural products, 3β-hydroxyspongia-13(16),14-dien-2-one (**15**) and 19-dehydroxy-spongian diterpene 17 (**16**), while the remaining five corresponded to previously reported terpenes, three spongia furanoditerpenes: 9-nor-3-hydroxyspongia-3,13(16)14-trien-2-one (**17**), 3β, 19 dihydroxyspongia-13(16),14-dien-2-one (epispongiadiol) (**18**), and spongian diterpene 17 (**19**); the furanoditerpene ambliol C (**20**) and the sesterterpene scalarin (**21**). The pharmacological analysis of the isolated compounds displayed a very mild cytotoxic activity for **15**, **18**, and **20**, while they showed no antimicrobial (*Acinetobacter baumannii, Pseudomonas aeruginosa, Klebsiella pneumoniae,* and *Staphylococcus aureus*) or antiviral (HAdV5 and HAdV5-GFP) activities [16] (Figure 4).

From the organic extracts of the brown seaweed *Dictyota ciliolata*, collected at the Caribbean coast of Quintana Roo state, Caamal-Fuentes et al*.* isolated the diterpenes pachydictyol A (**22**) and dictyol B acetate (**23**) in 2014. Cytotoxic and antiproliferative activities of the isolated compounds were evaluated on a panel of cancer cell lines (oral carcinoma (KB), epithelial carcinoma of the larynx (Hep-2), breast adenocarcinoma (MCF-7), and cervix adenocarcinoma (SiHa)) and a human cell embryonic kidney cell line HEK-293 as the control). Compound **22** exhibited inhibitory activity against all of the tested cancer cell lines, whereas diterpene **23** showed cytotoxic activity against epithelial carcinoma of the larynx-HEP-2 (CC_50_ = 19.6 μg mL^−1^/0.056 µM) and antiproliferative activity against breast-MCF-7 (IC_50_ = 38.3 μg mL^−1^/0.11 µM) and cervix-SiHa (IC_50_ = 34.4 μg mL^−1^/0.099 µM) [17] (Figure 4).

#### 2.2.2. Steroids

**Figure 5:**
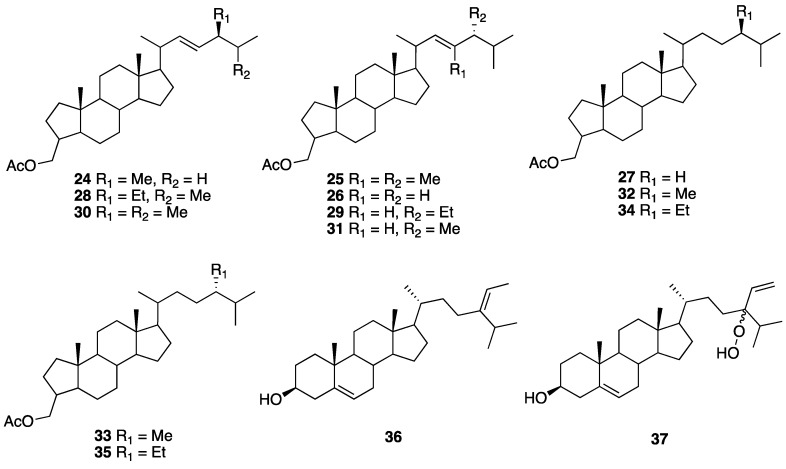
Steroid structures of the A-nor-5α-cholestanes **24**–**35** isolated from the sponge *Teichaxinella morchella* (now *Axinella corrugata*) and the cholesterol derivatives **36** and **37** isolated from the brown algae *Padina sanctae-crucis* and *Turbinaria tricostata*.

High diversity unusual structures of steroid derivatives with multiple potential biological properties have been isolated from marine organisms. Bohlin et al. reported several marine steroids as acetates from the sponge *Teichaxinella morchella* (now *Axinella corrugata*) collected at a depth of 15 m at Puerto Morelos in Quintana Roo state in 1981. Two new sterols, (22*E*,24*S*)-3*E*-acetoxymethyl-24-methyl-27-nor-A-nor-5α-cholest-22-ene (A-nor-patinosterol) (**24**) and (22*E*,24*R*)-3*E-*acetoxymethyl-23, 24-dimethyl-A-nor-5α-cholest-22-ene (A-nor-dinosterol) (**25**), along with six known sterols, (22*E*)-3*E*-acetoxymethyl-A-nor-5α-cholest-22-ene) (**26**), 3*E*-acetoxymethyl-A-nor-5α-cholestane (**27**), (22*E*,24*S*)-3*E*-acetoxymethyl-24-ethyl-A-nor-5α-cholest-22-ene (**28**), (22*E*,24*R*)-3*E*-acetoxymethyl-24-ethyl-A-nor-5α-cholest-22-ene (**29**), (22*E*,24*S*)-3*E*-acetoxymetyl-24-methyl-A-nor-5α-cholest-22-ene (**30**), and (22*E*,24*R*)-3*E*-acetoxymethyl-24-methyl-A-nor-5α-colest-22-ene (**31**) were isolated. Furthermore, four known steroids were also detected, (24*S*)-3*E*-acetoxymethyl-24-methyl-A-nor-5α-cholestane (**32**), (24*R*)-3*E*-acetoxymethyl-24-methyl-A-nor-5α-cholestane (**33**), (24*R*)-3*E*-acetoxymethyl-24-ethyl-A-nor-5α-cholestane (**34**), and (24*S*)-3*E*-acetoxymethyl-24-ethyl-A-nor-5α-cholestane (**35**) [18]. The relative configuration at C-3 and C-20 of **24**–**35** was not specified (Figure 5), and no biological data were reported for these compounds.

From two brown algae, *Padina sanctae-crucis* and *Turbinaria tricostata*, which were collected at the Caribbean coast of Quintana Roo state, were reported in 2014 from the isolation of fucosterol (**36**) and 24*E*-hydroperoxy-24-vinylcholesterol (**37**). Cytotoxic (CC_50_) and antiproliferative (IC_50_) activity assays on a panel of human cancer cell lines (KB, Hep-2, MCF-7, and SiHa) and a human cell embryonic kidney cell line HEK-293 as the control, showed that **36** is cytotoxic against Hep-2 and SiHa cell lines (CC_50_ of 14.8 and 18.6 μg mL^−1^/0.036 and 0.045 µM, respectively), with a high selectivity index towards Hep-2 (SI = 10) and antiproliferative activity against MCF-7 and SiHa (IC_50_ of 43.3 and 34.0 µg/mL/0.10 and 0.083 µM, respectively). Fucosterol (**36**) was also isolated from the brown algae *Dictyota ciliolata*. Steroid **37** displayed not only the highest cytotoxic activity (CC_50_ of 3.1 μg mL^−1^/7.0 nM), but also a high selectivity index (SI = 16.2) on KB cell lines. Additionally, **37** exhibited a moderate cytotoxic activity towards the Hep-2, MCF-7, and SiHa cell lines (CC_50_ of 10.5, 12.1, and 18.9 μg mL^−1^/0.024, 0.027, and 0.042 µM, respectively) with a lower selectivity index (SI of 4.7, 4.1, and 12.6, respectively) [17] (Figure 5).

#### 2.2.3. Triterpenoid Saponins

**Figure 6:**
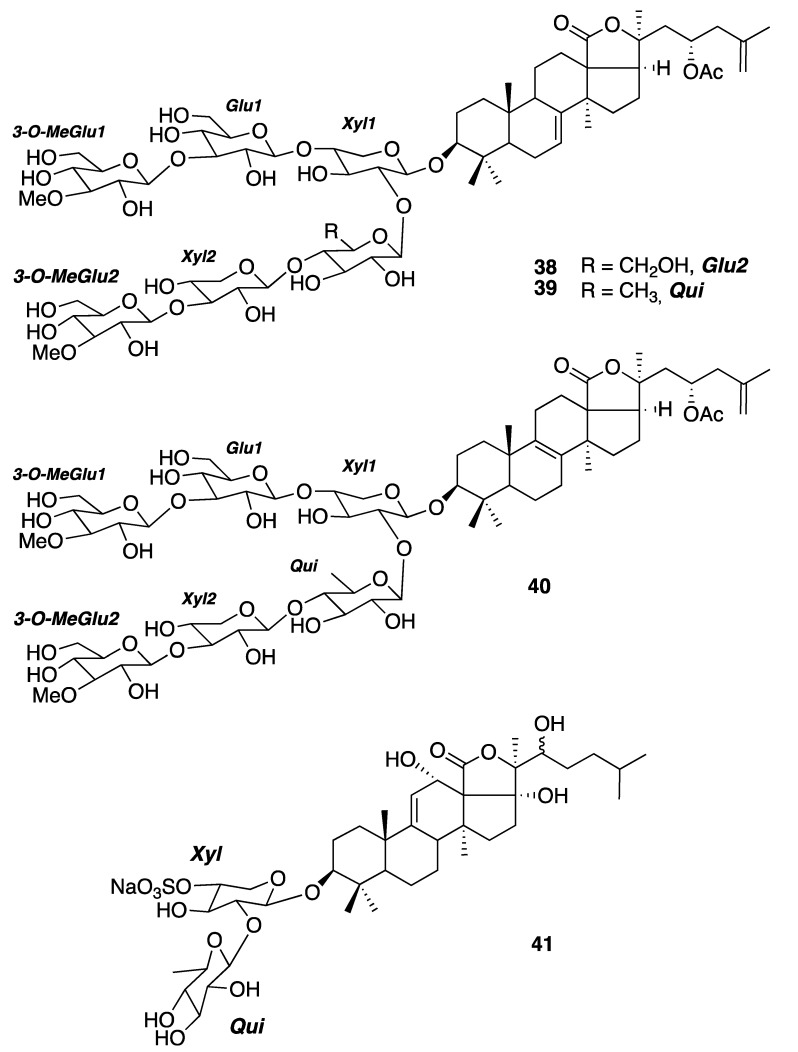
Structures of the triterpenoid saponins isolated from the sea cucumbers: the hexaglycosides **38**–**40** from *Astichopus multifidus* and the diglycoside **41** from *Holothuria floridana* (now *H.* (*Halodeima*) *floridana*).

Sea cucumbers constitute a rich source of triterpenoid saponins, with some of them exerting pharmacological effects [19]. From the sea cucumber *Astichopus multifidus*, collected on the Yucatan Peninsula coasts, Mena-Rejón and collaborators reported the isolation and the structural elucidation of three oligoglycoside triterpenes in 2016. Two of them, stichloroside B_2_ (**38**) and astichoposide C (**39**), were known, while the third one, named as astichoposide D (**40**), turned out to be a new natural product. Antiproliferative activity assays against two cancer lines, MCF-7 (ATCC HTB-22) and a highly invasive triple-negative breast cancer MDA-MB-231 (ATCC HTB-26), displayed that 38 had the highest antiproliferative activity against MCF-7 cells (6.45 μM), while 39 had the highest antiproliferative activity against the MDA-MB-231 cells (3.80 μM) [20]. The research group of Mena-Rejón also reported in 2013 the isolation of the known triterpenoid saponin holothurin B_2_ (**41**) from the sea cucumber *Holothuria floridana* (now *H.* (*Halodeima*) *floridana*) [21] (Figure 6).

### 2.3. Nitrogen Compounds

#### 2.3.1. Indole Derivatives

**Figure 7:**
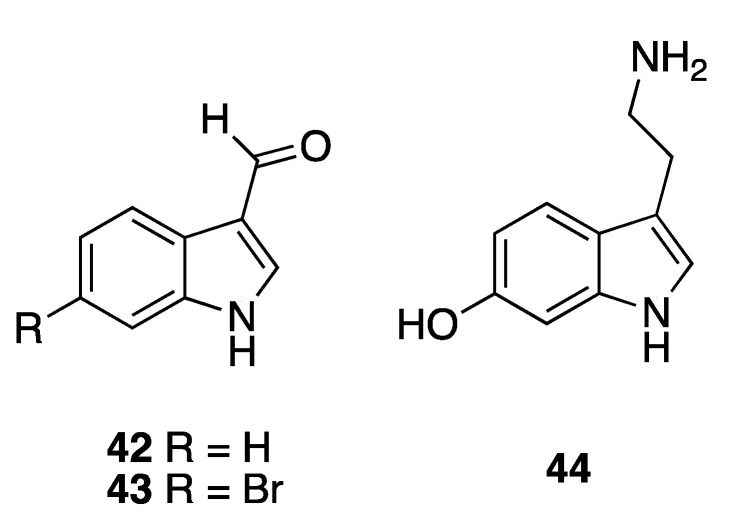
Structures of indole derivatives **42** and **43** from the tunicate *Stomozoa murrayi* (now *Stomozoa roseola*) and the bacterium *Acinetobacter* sp. and **44** from the mollusk *Octopus maya*.

Olguin-Uribe et al. isolated two indoles, indole-3-carbaldehyde (**42**) and its brominated derivate, 6-bromoindole-3-carbaldehyde (**43**), in 1997 from two different sources, the tunicate *Stomozoa murrayi* (currently known as *Stomozoa roseola*) and the bacterium *Acinetobacter* sp. associated to its surface [22]. The tunicate was collected at a depth of 3–5 m in Puerto Morelos, Quintana Roo state, very close to the Institute of Marine Sciences and Limnology research station of the National Autonomous University of Mexico (UNAM). These compounds were evaluated in several biological assays. The brominated indole **43** displays antimicrobial activity by inhibiting the growth of four marine bacterial strains SM-S2, SM-Z, *Bacillus marinus*, and *Vibrio campbellii*, while its debrominated analog **42** shows no inhibitory activity. On the other hand, both of the compounds exhibit antifouling activity by completely inhibiting the settlement of *Balanus amphitrite* (now Amphibalanus amphitrite) at the highest concentration tested at 100 μg mL^−1^ (0.13 and 0.084 µM respectively) and, even, the most active compound **43** can inhibit larval settlement by 80% at 10 μg mL^−1^/0.044 µM. Finally, these compounds showed no antipredatory (deterrent) activity against the *Serranus cabrilla* fish, which were collected in the Mediterranean Sea, or significant antialgal activity against the diatom *Nitzchia acicularis* (Figure 7).

Pech-Puch et al. reported the isolation of another indole, serotonin (**44**), from the salivary glands of *Octopus maya* collected in Sisal, Yucatan state in 2016 [23]. The neurotoxic activity previously found in its extract was attributed to that compound (Figure 7).

#### 2.3.2. Nucleosides and Nitrogenous Bases

The importance of the study of nucleosides comes from the fact that the arabino-nucleosides spongothymidine and spongouridine, isolated from a marine sponge, were the first marine natural products that showed their potential as drugs, because they constituted the basis of the development of the first synthetic nucleosides approved as therapeutic drugs: the anticancer cytarabine (*ara*-C) and the antiviral vidarabine (*ara*-A) [1].

**Figure 8:**
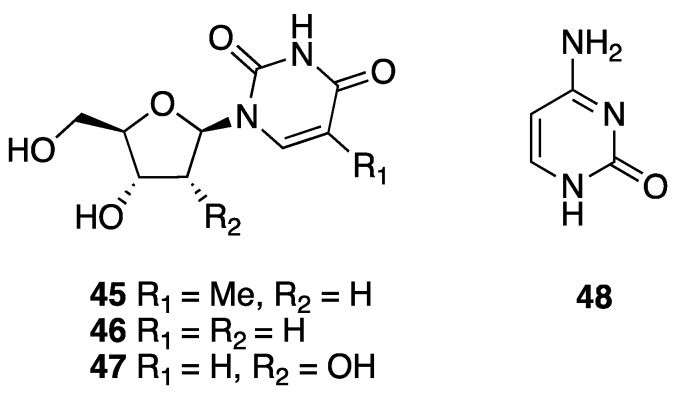
Structures of nucleosides **45**–**47** and nitrogenous base **48** isolated from the sponges *Halichondria magniconulosa* (now *H.* (*Halichondria*) *magniconulosa*) and *Haliclona tubifera* (now *H.* (*Reniera*) *tubifera*), respectively.

Three nucleosides, thymidine (**45**), 2′-desoxyuridine (**46**), and uridine (**47**), were isolated from the sponge *Halichondria magniconulosa* (now *H.* (*Halichondria*) *magniconulosa*) collected at 0.5–1 m of depth in Chabihau, Yucatan state, and reported by the research group of Mena-Rejón in 2018 [24]. The same year, Medina-Gómez et al. reported the isolation of cytosine (**48**) from the sponge *Haliclona tubifera* (now *H.* (*Reniera*) *tubifera*) [12] (Figure 8). No biological data were reported for **45**–**48**.

#### 2.3.3. Conotoxins

The venomous fish-hunting cone snails that belong to the *Conus* genus are composed of a collection of toxin peptides that serve to immobilize prey by targeting different physiological mechanisms in their neuromuscular system. In this way, ω-conotoxin MVIIA, isolated from *Conus magus*, is commercialized as the synthetic Prialt^®^ (ziconatide), which constituted the first FDA-approved drug that was directly derived from a marine natural product as a pain control drug [1]. On the basis of this background, the research group lead by Aguilar from the Institute of Neurobiology-UNAM at Queretaro in Mexico, reported the isolation of new conotoxins from three different snails, mainly belonging to the *Conus* genus, which were collected along the coasts of the Mexican Caribbean.

**Figure 9:**
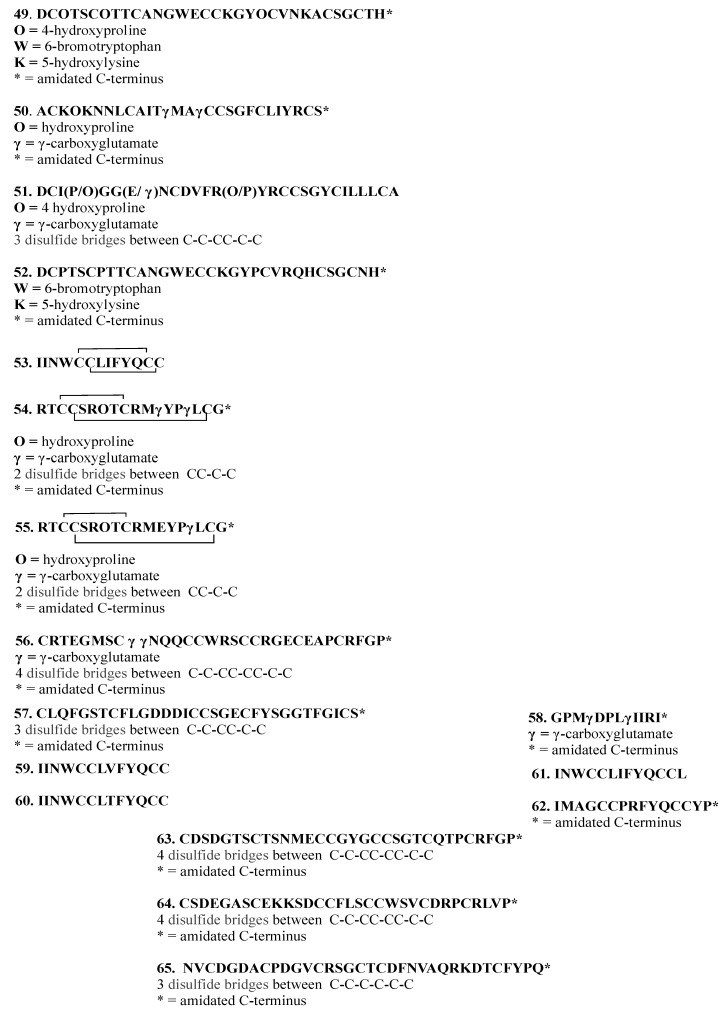
Structures of conotoxins **49**–**65** isolated from cone snails belonging to the *Conus* genus and *Polystira albida*.

Thus, the study of the extract of the venomous duct of *Conus delessertii* (now *Conasprella delessertii*), mollusk collected in Quintana Roo state, allowed Aguilar and collaborators to report four new peptides: conotoxin de13a (**49**) and conotoxin de7a (**50**), reported in 2005, conotoxin de7b (**51**), reported in 2009, and conotoxin de13b (**52**), reported in 2013. Conotoxin de13a (**49**) contains 32 amino acids (3486.76 Da) and it was defined as a new class of conotoxins. This peptide is characterized by the presence of high content of post-translational modified amino acids, such as 5-hydroxylysine, and the residues of cysteine arranged in a pattern (C-C-C-CC-C-C-C), which were not previously described in conotoxins [25]. On the other hand, conotoxin de7a (**50**) contains 28 amino acids (3170.0 Da), with some of them being post-transductionally modified, and a residue (-γCCS-) previously found only in two other conotoxins [26]. Conotoxin de7b (**51**), bearing 28 amino acids, including six cysteine residues, is characterized by existing in different post-transductional modified isomorphs (with molecular masses varying from 3078.6 to 3154.6 Da), some of them containing γ-carboxy-glutamate and/or 4-hydroxyproline at positions 4, 7, and/or 14 [27]. Finally, conotoxin de13b (**52**) has the same arrangement of cysteine residues as conotoxin de13a (**49**) [28] (Figure 9).

From the extract of the venom duct of a second mollusk, *Conus spurius*, Aguilar and collaborators reported the isolation of twelve new conotoxin derivatives **53**–**64**, from two different places: Quintana Roo and Campeche states. Conotoxin sr5a (**53**), which was reported in 2006, is a hydrophobic peptide belonging to the T-1 conotoxin family with a molecular mass of 1616.60 Da and a pair of disulfide bridges. In a biological test in mice, this conotoxin caused a depressed behavioral activity [29]. One year later, two new α-conotoxins of 18 amino acids, SrIA (**54**) and SrIB (**55**), with a molecular mass of 2202.9 and 2158.8 Da, respectively, were reported. Conotoxis **54** and **55** were evaluated as antagonists to nicotinic acetylcholine receptors in order to search for new therapeutic alternatives against brain diseases (schizophrenia, nocturnal frontal lobe epilepsy and Alzheimer’s disease). The results suggested not only that these conotoxins can operate as nicotinic acetylcholine receptor inhibitors, but also that they bind to nicotinic acetylcholine receptors with a very high affinity, increasing their intrinsic cholinergic response, and making them excellent model tools for studying toxin-receptor interaction [30]. The fourth new peptide, conotoxin sr11a (**56**), with a molecular weight of 3650.77 Da, was reported in 2007, being the first I-conotoxin that was isolated from the Western Atlantic. This peptide produces a stiffening of body, limbs, and tail when intracranially injected into mice [31]. Conotoxin sr7a (**57**), containing 32 amino acids (3330.74 Da) and reported in 2007, displays several in vivo effects, such as hyperactivity in mice and paralysis in freshwater snails (*Pomacea paludosa*), while it was inactive in intramuscular trials with the limpet *Patella opea* and the freshwater fish *Lebistes reticulatus* [32]. In contrast, conorfamide-Sr2 (CNF-Sr2, **58**), as reported in 2008, with a molecular mass of 1468.70 Da and without cysteine residues, exhibits paralytic activity in the limpet *Patella opea* and produces hyperactivity in the freshwater snail *Pomacea paludosa* and mice [33]. From specimens of *Conus spurius*, collected in Isla Arena, Campeche state, were isolated and identified by reverse transcription polymerase chain reaction, seven conotoxins. Four of them belong to the T-1 conotoxin family, (18V) sr5a (**59**), (18T) sr5a (**60**), “extended” (**61**), and “hydrophilic” (**62**), which were reported in 2009 [34], and they are very similar to the conotoxin sr5a (**53**). The other three, reported in 2010, were the known conotoxin sr11a (**56**) already reported in 2007 [31] and the new conotoxins, sr11b (**63**) and sr11c (**64**) [35] (Figure 9).

Finally, Aguilar and collaborators reported in 2009 the isolation of a new peptide, pal9a (**65**) (3678.84 Da) with 34 amino acids, including six cysteine residues, from a third mollusk, *Polystira albida*, collected in Campeche state. This is the first P-conotoxin-like turritoxin isolated from a member of the family Turridae from the Western Atlantic [36] (Figure 9).

### 2.4. Biopolymers

**Figure 10:**
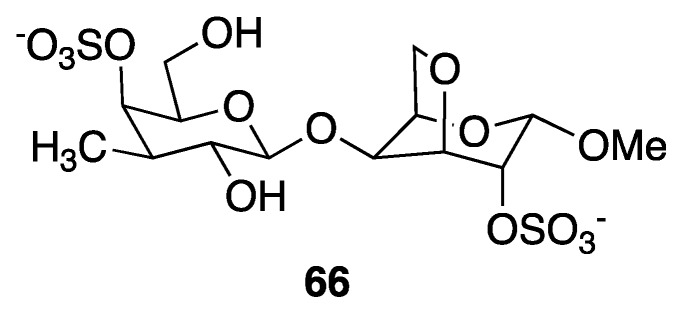
Structure of L-carrageenan isolated from the red algae *Solieria filiformis*.

Freile-Pelegrín and collaborators reported the characterization of l-carrageenan (**66**) in 2018, being obtained from the direct extraction of the red algae *Solieria filiformis* collected at Telchac in the Yucatan state. This polysaccharide shows high antiviral activity against Herpes simplex virus with an EC_50_ value of 6.3 μg mL^−1^/0.019 μM [37] (Figure 10).

## 3. Bioprospecting Overview

**Figure 11:**
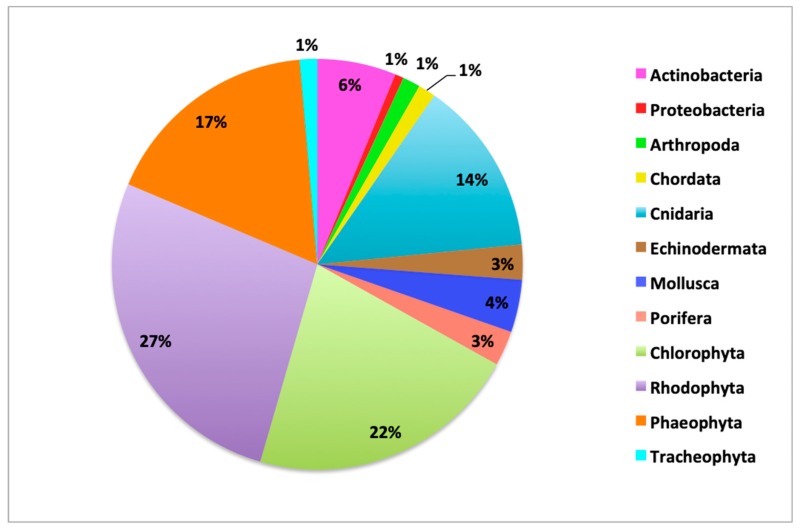
Distribution of the reported marine organisms by phylum.

A total of 95 scientific documents were recorded and analyzed in this review, including 82 articles, four postgraduate dissertation theses, and nine meeting abstract communications. They describe the reports related to research on pharmacological surveys of extracts, chemical composition, and isolation of marine natural products. A total of 145 species of marine organisms are enclosed, belonging to 12 phyla (Table 1 and Table 2), being the most representative Rhodophyta (27%), Chlorophyta (22%), Phaeophyta (17%), and Cnidaria (14%) (Figure 11).

**Figure 12:**
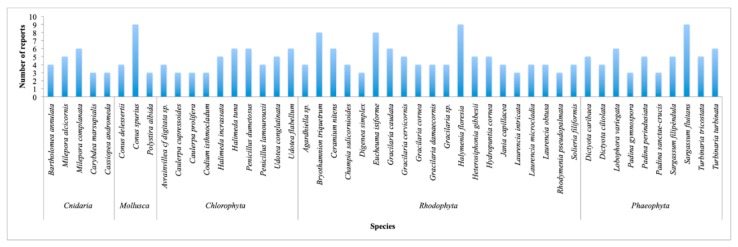
Number of publications of reported marine species organized by phylum. Only those species that have three or more reports are displayed.

The mollusk *Conus spurius* and two algae, *Halymenia floresia* (now *H. floresii*) and *Sargassum fluitans*, with nine reports each, were the most reported species. The coral *Millepora complanata* and eight algae, *Halimeda tuna*, *Penicillus dumetosus*, *Udotea flabellum*, *Bryothamnion triquetrum* (now *Alsidium triquetrum*), *Ceramium nitens*, *Eucheuma isiforme* (now *Eucheumatopsis isiformis*), *Gracilaria caudata* (now *Crassiphycus caudatus*), *Lobophora variegata*, and *Turbinaria turbinata*, with 6–8 reports each, following the list as it is shown in Figure 12.

**Figure 13:**
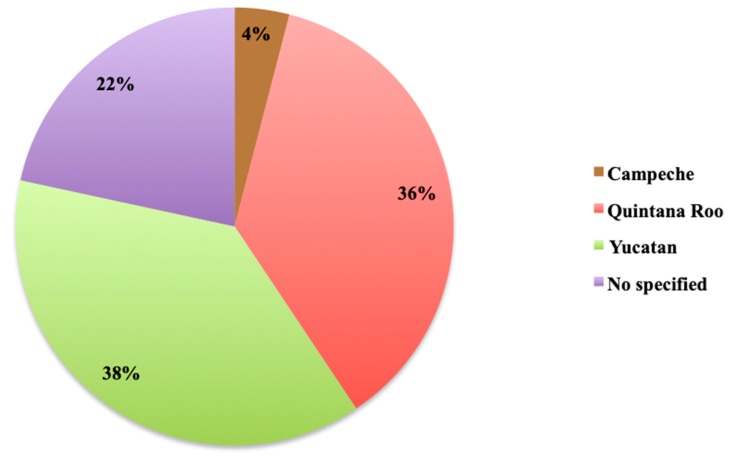
Geographic distribution of collections sources in percentage by state.

From the territorial distribution point of view, the highest number of reports corresponds to marine organisms that were collected at the coast of the Yucatan state (38%), followed by the coasts of Quintana Roo state (36%) and, finally, the coasts of Campeche state (4%). However, 22% of the reports did not specify the state where the marine organisms were collected (Figure 13).

**Figure 14:**
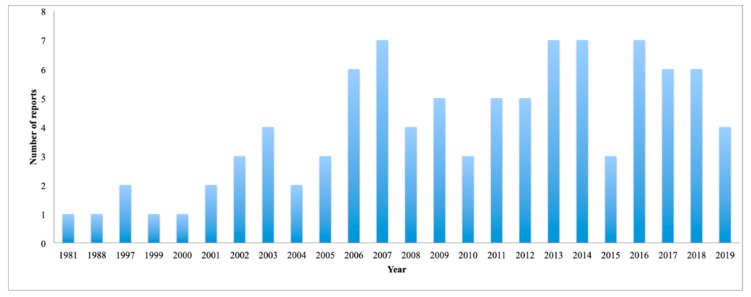
Number of publications per year.

Figure 14 displays the number of reports per year. As far as we know, the first report was published in 1981 and, since then, the number of publications related to the search for natural marine products of the Yucatan Peninsula has been increasing. However, this increase was not constant, being the years 2007, 2013, 2014, and 2016, with seven publications each, when more reports were published.

## 4. Conclusions

The present review represents the first comprehensive report of natural products that have been isolated from marine organisms collected along the coasts of the Yucatan Peninsula, covering literature up to mid-2019. As result of 38 years of investigations of marine organisms that were collected in the Yucatan Peninsula, 66 marine natural products were isolated from 18 species belonging to eight different phyla (Proteobacteria (*Acinetobacter* sp.), Chordata (ascidian: *Stomozoa murrayi* (now *Stomozoa roseola*), Cnidaria (*Pterogorgia anceps*), Echinodermata (*Astichopus multifidus* and *Holothuria floridana* (now *H.* (*Halodeima*) *floridana*)), Mollusca (*Conus delessertii* (now*Conasprella delessertii*), *Conus spurius*, *Octopus maya,* and *Polystira albida*), Porifera (*Halichondria magniconulosa* (now *H.* (*Halichondria*) *magniconulosa*), *Haliclona tubifera* (now *H.* (*Reniera*) *tubifera*), *Spongia tubulifera* (now *S.* (*Spongia*) *tubulifera*) and *Teichaxinella morcella* (now *Axinella corrugata*)), Rhodophyta (*Solieria filiformis*), and Phaeophyta (*Dictyota ciliolata, Lobophora variegata*, *Padina sanctae-crucis,* and *Turbinaria tricostata*) (Table 1). Out of the 66 marine natural products identified, 26 correspond to structures that were not previously reported. These 26 new chemical entities correspond to three aliphatic polyketides (**1**–**3**), one glycolipid (**7**), two diterpenes (**15**, **16**), two steroids (**24**, **25**), one triterpenoid saponin (**40**), and 17 conotoxins (**49**–**65**).

**Figure 15:**
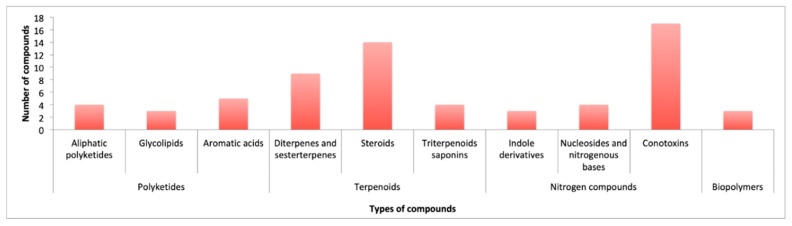
Biosynthetic classes of the reported marine natural products.

Figure 15 displays the overall biogenetic distribution of the reported compounds. The terpenoid biogenesis is again the most prominent pathway (40.9%), enclosing the diterpenes-sesterterpenes (nine compounds), steroids (14 compounds), and triterpenoids saponins (four compounds). On the other hand, conotoxins, with 17 compounds (25.8%) constitute the largest group of the reported natural products.

The biological studies of the isolated compounds are focused on cytotoxic or antiproliferative activities (diterpenes **15**, **18**, **20, 22**, and **23**; steroids **36** and **37**, and triterpenoids saponins **38** and **39**), the antimicrobial, antifouling, antipredatory (deterrent), and antialgal activity (indole derivative **42** and **43**), the antiprotozoal activity (glycolipids **7**–**9**), neurotoxic activity (indole derivative **44**), behavioral activity in animal models (conotoxins **53**, **56**–**58**), and finally in the interesting pharmacological activities against brain diseases of the new conotoxins **54** and **55**, and the high antiviral activity of the known biopolymer L-carrageenan (**66**).

As a concluding remark, this review shows the potential of the Yucatan Peninsula as an interesting source of new marine natural products, not only because of its unique and rich diversity of marine organisms, but also due to the small number of works that have been published so far, which indicates that this area of research has been poorly investigated. For these reasons, the marine biodiversity of the Yucatan Peninsula can be considered as a poor exploited source of new bioactive marine natural products, which could be the base of the development of new drugs.

## Figures and Tables

**Table 1:** Reported marine species of the Yucatan Peninsula in which natural products were isolated.

Phylum	Species	Compounds Isolated and Biogenetic Origin	References
Proteobacteria	*Acinetobacter* sp.	**42**, **43** (Nitrogen compounds)	[22]
Chordata (Ascidian)	*Stomozoa murrayi* (now *Stomozoa roseola*)	**42**, **43** (Nitrogen compounds)	[22]
Cnidaria (Coral)	*Pterogorgia anceps*	**1**–**3** (Polyketides)	[11]
Echinodermata (Sea cucumbers)	*Astichopus multifidus*	**38**–**40** (Terpenoids)	[20]
	*Holothuria floridana* (now *H.* (*Halodeima*) *floridana*)	**41** (Terpenoid)	[21]
Mollusca (Mollusks)	*Conus delessertii* (now *Conasprella delessertii*)	**49**–**52** (Nitrogen compounds)	[25,26,27,28]
	*Conus spu* *rius*	**53**–**64** (Nitrogen compounds)	[29,30,31,32,33,34,35]
	*Octopus maya*	**44** (Nitrogen compound)	[23]
	*Polystira albida*	**65** (Nitrogen compound)	[36]
Porifera (Sponges)	*Halichondria magniconulosa* (now *H.* (*Halichondria*) *magniconulosa*)	**45**–**47** (Nitrogen compounds)	[24]
	*Haliclona tubifera* (now *H.* (*Reniera*) *tubifera*)	**6**, **10**–**14** (Polyketides) and **48** (Nitrogen compound)	[12]
	*Spongia tubulifera* (now *S.* (*Spongia*) *tubulifera*)	**15**–**21** (Terpenoids)	[16]
	*Teichaxinella morchella* (now *Axinella corrugata*)	**24**–**35** (Terpenoids)	[18]
Phaeophyta (Brown algae)	*Dictyota ciliolata*	**22**, **23**, **36** (Terpenoids)	[17]
	*Lobophora variegata*	**7**–**9** (Polyketides)	[14]
	*Padina sanctae-crucis*	**36**, **37** (Terpenoids)	[17]
	*Turbinaria tricostata*	**36**, **37** (Terpenoids)	[17]
Rhodophyta (Red algae)	*Solieria filiformis*	**66** (Biopolymer)	[37]

**Table 2:** Reported marine species from Yucatan Peninsula related to bioprospecting without determining the chemical composition.

**Domain:** Bacteria		
**Kingdom:** Bacteria		
**Phylum**	**Genus or Species**	**References**
Actinobacteria	*Streptomyces*	[38]
	*Saccharomonospora*	
	*Dietzia*	
	*Nocardiopsis*	
	*Pseudonocardia*	
	*Verrucosispora*	
	*Brachybacterium*	
	*Jiangella*	
	*Salinispora*	
**Domain:** Eukarya		
**Kingdom:** Animalia		
Arthropoda (Crustaceans)	*Bathynomus giganteus*	[39]
	*Limulus Polyphemus*	[40]
Chordata (Ascidians)	*Trididemnum solidum*	[41]
Cnidaria (Anemones)	*Actiinidae* Gen. sp. nov *.	[42]
	*Anthopleura texaensis*	
	*Bartholomea annulata*	[43,44,45,46]
	*Bunodeopsis antilliensis*	[47]
	*Bunodeopsis globulifera* (now *Viatrix globulifera*)	[48,49]
	*Condylactis gigantea*	[50]
	*Lebrunia danae* (now *L. neglecta*)	[47,51]
	*Stichodactyla helianthus*	[47,52]
	Telmatactis bernoni*	[43]
Cnidaria (Corals)	*Millepora alcicornis*	[53,54,55,56,57]
	*Millepora complanata*	[53,55,58,59,60,61,62]
	*Porites astreoides*	[63]
	*Pseudodiploria strigosa*	[63]
	*Siderastrea siderea*	[63]
Cnidaria (Jellyfish)	*Aurelia aurita*	[64,65]
	*Carybdea marsupialis*	[47,66,67]
	*Cassiopea xamachana* (now *Cassiopea andromeda*)	[47,68,69]
	*Linuche unguiculata*	[47]
	*Pelagia noctiluca*	[70]
Echinodermata (Sea cucumbers)	*Holothuria mexicana* (now *H*. (*Halodeima*) *mexicana*)	[71]
	*Isostichopus badionotus*	[72,73,74]
Mollusca (Mollusks)	*Conus austini* (now *C. cancellatus*)	[75]
	*Conus spu* *rius*	[75,76]
	*Gemmula periscelida*	[77]
	*Octopus maya*	[78]
	*Polystira albida*	[75,77]
**Domain:** Eukarya		
**Kingdom:** Plantae		
Chlorophyta (Green algae)	*Acetabularia schenckii* (now *A. subg. Acicularia schenckii*)	[79]
	*Avrainvillea* cf *digitata* sp.	[80,81,82,83]
	*Avrainvillea longicaulis*	[79]
	*Avrainvillea nigricans*	[84]
	*Caulerpa ashmeadii*	[79,85]
	*Caulerpa cupressoides*	[79,85,86]
	*Caulerpa mexicana*	[85]
	*Caulerpa paspaloides*	[79,85]
	*Caulerpa prolifera*	[79,85,87]
	*Caulerpa racemosa*	[85]
	*Caulerpa racemosa var. racemosa*	[88]
	*Caulerpa sertularioides*	[79,87]
	*Caulerpa taxifolia*	[79]
	*Cladophora prolifera*	[79]
	*Cladophora vagabunda*	[79]
	*Codium decorticatum*	[79,83]
	*Codium isthmocladum*	[85,88,89]
	*Enteromorpha intestinalis* (now *Ulva intestinalis*)	[79]
	*Halimeda incrassata*	[80,81,82,83,85]
	*Halimeda monile*	[79]
	*Halimeda opuntia*	[86]
	*Halimeda tuna*	[79,80,81,82,83,86]
	*Penicillus capitatus*	[85,86]
	*Penicillus dumetosus*	[79,80,81,82,83,90]
	*Penicillus lamourouxii*	[80,81,82,83]
	*Penicillus pyriformis*	[79]
	*Rhipocephalus phoenix*	[81,82]
	*Rhipocephalus phoenix f brevifolius*	[80,83]
	*Udotea conglutinata*	[79,80,81,82,83]
	*Udotea flabellum*	[80,81,82,83,86,91]
	*Udotea occidentalis*	[85]
Phaeophyta (Brown algae)	*Dictyopteris jamaicensis*	[83]
	*Dictyota bartayresiana*	[86]
	*Dictyota caribaea*	[80,81,82,83,92]
	*Dictyota cervicornis* (now *Canistrocarpus cervicornis*)	[79,86]
	*Dictyota ciliolata*	[79,93,94]
	*Dictyota crenulata*	[79]
	*Dictyota dichotoma*	[89]
	*Dictyota menstrualis*	[85]
	*Lobophora variegata*	[79,80,81,82,83]
	*Padina boergesenii*	[86]
	Padina durvillaei*	[86]
	*Padina gymnospora*	[79,86,88]
	*Padina pavonica*	[83]
	*Padina perindusiata*	[80,81,82,83,92]
	*Padina sanctae-crucis*	[93,94]
	*Sargassum cymosun*	[86]
	*Sargassum filipendula*	[84,85,86,88,92]
	*Sargassum fluitans*	[80,81,82,83,86,93,94,95,96]
	*Sargassum hystrix*	[84,86]
	*Sargassum polyceratium*	[86]
	*Sargassum pteropleuron*	[79,86]
	*Sargassum ramifolium*	[79]
	*Sargassum vulgare*	[86]
	*Turbinaria tricostata*	[79,86,93,97]
	*Turbinaria turbinata*	[80,81,82,83,86,92]
Rhodophyta (Red algae)	*Acanthophora spicifera*	[79]
	*Agardhiella* sp.	[80,81,82,83]
	*Agardhiella subulata*	[89]
	*Bryothamnion triquetrum* (now *Alsidium triquetrum*)	[79,80,81,82,83,85,86,89]
	*Ceramium nitens*	[79,80,81,82,83,85]
	*Champia salicornioides*	[79,80,82,83]
	*Chondria atropurpurea*	[79]
	*Chondria baileyana*	[79]
	*Chondrophycus papillosus* (now *Palisada perforata*)	[79]
	*Chondrophycus poiteaui* (now *Yuzurua poteaui*)	[79]
	*Digenea simplex*	[79,85,86]
	*Eucheuma isiforme* (now *Eucheumatopsis isiformis*)	[79,80,81,82,83,85,88,98]
	*Gracilaria blodgettii*	[99]
	*Gracilaria bursa-pastoris*	[79]
	*Gracilaria caudata* (now *Crassiphycus caudatus*)	[79,80,81,82,83,85]
	*Gracilaria cervicornis*	[80,81,82,83,99]
	*Gracilaria cornea* (now *Crassiphycus corneus*)	[79,85,88,100]
	*Gracilaria crassissima* (now *Crassiphycus crassissimus*)	[99,100]
	*Gracilaria cylindrica*	[79]
	Gracilaria damaecornis*	[80,81,82,83]
	*Gracilaria* sp.	[80,81,82,83]
	*Gracilaria tikvahiae*	[79]
	*Gracilariopsis tenuifrons*	[79,101]
	*Halymenia floresia* (now *H. floresii*)	[79,80,82,83,84,85,89,102,103]
	*Heterosiphonia gibbesii*	[79,80,81,82,83]
	*Hydropuntia cornea* (now *Crassiphycus corneus*)	[80,81,82,83,95]
	*Hypnea musciformis*	[104]
	*Hypnea spinella*	[79]
	*Jania capillacea*	[80,81,82,83]
	*Laurencia intricata*	[79,86,87]
	*Laurencia microcladia*	[80,81,82,83]
	*Laurencia obtusa*	[79,84,85,86]
	*Laurencia papillosa* (now *Palisada perforata*)	[86]
	*Laurencia poiteaui* (now *Palisada poiteaui*)	[85,86]
	*Liagora ceranoides*	[79]
	Nemalion helmintoides*	[79]
	*Rhodymenia pseudopalmata*	[95,105,106]
	*Solieria filiformis*	[95,107,108]
	*Spyridia filamentosa*	[87]
Tracheophyta (Seagrasses)	*Syringodium filiforme*	[87]
	*Thalassia testudinum*	[87]

* Organisms not found in World Register of Marine Species (WORMS database) [109].

## References

[1] Jiménez C. (2018). Marine Natural Products in Medicinal Chemistry. ACS Med. Chem. Lett..

[2] Altmann K.H. (2017). Drugs from the Oceans: Marine Natural Products as Leads for Drug Discovery. Chimia.

[3] Mayer A.M.S., Rodríguez A.D., Taglialatela-Scafati O., Fusetani N. (2017). Marine Pharmacology in 2012–2013: Marine Compounds with Antibacterial, Antidiabetic, Antifungal, Anti-Inflammatory, Antiprotozoal, Antituberculosis, and Antiviral Activities; Affecting the Immune and Nervous Systems, and Other Miscellaneous Mechanisms of Action. Mar. Drugs.

[4] Barrera A. (1962). La Península de Yucatán Como Provincia Biótica. Rev. Soc. Mex. Hist. Nat..

[5] Hernández-Bolio G.I., Ruiz-Vargas J.A., Peña-Rodríguez L.M. (2019). Natural Products from the Yucatecan Flora: Structural Diversity and Biological Activity. J. Nat. Prod..

[6] Pech Pool D., Ardisson Herrera P.L., Duran R., Méndez M. (2010). Diversidad en el Bentos Marino-Costero. Biodiversidad y Desarrollo Humano en Yucatán.

[7] Garza Pérez J.R., Simões N., Chiappa Carrara X., Cucio C., Mascaró Miquelajáuregui M., Oseguera Cruz M., Lozano Aburto M., Acosta González G., Duran R., Méndez M. (2010). Comunidades Coralinas de las Bajas de Sisal. Biodiversidad y Desarrollo Humano en Yucatán.

[8] Torruco Gómez D., González Solís A., Duran R., Méndez M. (2010). Las Esponjas y Su Importancia. Biodiversidad y Desarrollo Humano en Yucatán.

[9] Acosta González G., Arias González J.E., Duran R., Méndez M. (2010). Comunidades Bentónicas Del Arrecife Alacranes. Biodiversidad y Desarrollo Humano en Yucatán.

[10] Palomino-Alvarez L.A., Moreira Rocha R., Simões N. (2019). Checklist of Ascidians (Chordata, Tunicata) from the Southern Gulf of Mexico. Zookeys.

[11] Guo Y.W., Gavagnin M., Mollo E., Trivellone E., Cimino G. (1999). Three New Butenolide Lipids from the Caribbean Gorgonian *Pterogorgia anceps*. J. Nat. Prod..

[12] Medina-Gómez S., Mirón-López G., Quijano-Quiñones R. (2018). Caracterización Estructural de Compuestos Obtenidos a Partir de Esponja de Mar Empleando Resonancia Magnética Nuclear y Cálculos Teóricos. 11° Foro en Ciencias Químicas y Bioquímicas.

[13] Fattorusso E., Mangoni A., Herz W., Kirby G.W., Moore R.E., Steglich W., Tamm C. (1997). Progress in the Chemistry of Organic Natural Products.

[14] Cantillo-Ciau Z., Moo-Puc R., Quijano L., Freile-Pelegrín Y. (2010). The Tropical Brown Alga *Lobophora variegata*: A Source of Antiprotozoal Compounds. Mar. Drugs.

[15] Carroll A.R., Copp B.R., Davis R.A., Keyzers R.A., Prinsep M.R. (2019). Marine Natural Products. Nat. Prod. Rep..

[16] Pech-Puch D., Rodríguez J., Cautain B., Sandoval-Castro C.A., Jiménez C. (2019). Cytotoxic Furanoditerpenes from the Sponge *Spongia tubulifera* Collected in the Mexican Caribbean. Mar. Drugs.

[17] Caamal-Fuentes E., Moo-Puc R., Freile-Pelegrín Y., Robledo D. (2014). Cytotoxic and Antiproliferative Constituents from *Dictyota ciliolata*, *Padina sanctae-crucis* and *Turbinaria tricostata*. Pharm. Biol..

[18] Bohlin L., Sjöstrand U., Djerassi C., Sullivan B. (1981). Minor and Trace Sterols in Marine Invertebrates. Part 20. 3E-Hydroxy-methyl-A-nor-Patinosterol and 3E-Hydroxymethyl-A-nor-dinosterol. Two New Sterols with Modified Nucleus and Side-Chain from the Sponge *Teichaxinella morchella*. J. Chem. Soc. Perkin Trans..

[19] Khotimchenko Y. (2018). Pharmacological Potential of Sea Cucumbers. Int. J. Mol. Sci..

[20] Graniel-Sabido M.J., Mirón-López G., León-Deniz L.V., Moo-Puc R.E., Quintal-Novelo C.J., Quijano L., Mena-Rejón G.J. (2016). Total NMR Assignment of a New Antiproliferative Triterpene Oligoglycoside from the Sea Cucumber *Astichopus multifidus*. Tetrahedron Lett..

[21] Salazar-Mendoza J., Padilla-Montaño N., León-Deniz L.V., Mena-Rejón G.J., Quijano L. (2013). Actividad Antifúngica de Metabolitos Aislados de la Pared Corporal de *Holoturia Floridana*. Foro en Ciencias Químicas y Bioquímicas. Posgrado Institucional en Ciencias Químicas y Bioquímicas.

[22] Olguin-Uribe G., Abou-Mansour E., Boulander A., Débard H., Francisco C., Combaut G. (1997). 6-Bromoindole-3-Carbaldehyde, from an *Acinetobacter* sp. Bacterium Associated with the Ascidian *Stomozoa murrayi*. J. Chem. Ecol..

[23] Pech-Puch D., Cruz-López H., Canche-Ek C., Campos-Espinosa G., García E., Mascaro M., Rosas C., Chávez-Velasco D., Rodríguez-Morales S. (2016). Chemical Tools of *Octopus maya* During Crab Predation Are Also Active on Conspecifics. PLoS ONE.

[24] Salazar Mendoza J., Mirón López G., Mena Rejón G.J. (2018). Estudio Químico de *Halichondria Magniconulosa* (Porifera: Demospongiae) del Litoral del Estado de Yucatán. 11° Foro en Ciencias Químicas y Bioquímicas.

[25] Aguilar M.B., López-Vera E., Ortiz E., Becerril B., Possani L.D., Olivera B.M., Heimer de la Cotera E.P. (2005). A Novel Conotoxin from *Conus delessertii* with Posttranslationally Modified Lysine Residues. Biochemistry.

[26] Aguilar M.B., López-Vera E., Imperial J.S., Falcón A., Olivera B.M., Heimer de la Cotera E.P. (2005). Putative γ-Conotoxins in Vermivorous Cone Snails: The Case of *Conus delessertii*. Peptides.

[27] Aguilar M.B., Flores-Torres A., Batista C.V.F., Falcón A., López-Vera E., Heimer de la Cotera E.P. (2009). Structural Characterization of Five Post-translationally Modified Isomorphs of a Novel Putative δ-Conotoxin from the Vermivorous Snail *Conus delessertii* from the Mexican Caribbean Sea. Peptides.

[28] Aguilar M.B., Ortiz E., Kaas Q., López-Vera E., Becerril B., Possani L.D., Heimer de la Cotera E.P. (2013). Precursor De13.1 from *Conus delessertii* Defines the Novel G Gene Superfamily. Peptides.

[29] Aguilar M.B., Lezama-Monfil L., Maillo M., Pedraza-Lara H., López-Vera E., Heimer de la Cotera E.P. (2006). A biologically active hydrophobic T-1-Conotoxin from the Venom of *Conus spurius*. Peptides.

[30] López-Vera E., Aguilar M.B., Schiavon E., Marinzi C., Ortiz E., Restano Cassulini R., Batista C.V.F., Possani L.D., Heimer de la Cotera E.P., Peri F. (2007). Novel α-Conotoxins from *Conus spurius* and the α-Conotoxin EI Share High-Affinity Potentiation and Low-Affinity Inhibition of Nicotinic Acetylcholine Receptors. FEBS J..

[31] Aguilar M.B., López-Vera E., Heimer de la Cotera E.P., Falcón A., Olivera B.M., Maillo M. (2007). I-Conotoxins in Vermivorous Species of the West Atlantic: Peptide Sr11a from *Conus spurius*. Peptides.

[32] Luna-Ramírez K.S., Aguilar M.B., Falcón A., Heimer de la Cotera E.P., Olivera B.M., Maillo M. (2007). An O-Conotoxin from the Vermivorous *Conus spurius* Active on Mice and Mollusks. Peptides.

[33] Aguilar M.B., Luna-Ramírez K.S., Echeverría D., Falcón A., Olivera B.M., Heimer de la Cotera E.P., Maillo M. (2008). Conorfamide-Sr2, a gamma-carboxyglutamate-containing FMRFamide-related Peptide from the Venom of *Conus spurius* with Activity in Mice and Mollusks. Peptides.

[34] Zamora-Bustillos R., Aguilar M.B., Falcón A., Heimer de la Cotera E.P. (2009). Identification, by RT-PCR, of Four Novel T-1-Superfamily Conotoxins from the Vermivorous Snail *Conus spurius* from the Gulf of Mexico. Peptides.

[35] Zamora-Bustillos R., Aguilar M.B., Falcón A. (2010). Identification, by Molecular Cloning, of a Novel Type of I_2_-Superfamily Conotoxin Precursor and Two Novel I_2_-Conotoxins from the Worm-Hunter Snail *Conus spurius* from the Gulf of México. Peptides.

[36] Aguilar M.B., Chan de la Rosa R.A., Falcón A., Olivera B.M., Heimer de la Cotera E.P. (2009). Peptide pal9a from the Venom of the Turrid Snail *Polystira Albida* from the Gulf of Mexico: Purification, Characterization, and Comparison with P-Conotoxin-like (framework IX) Conoidean Peptides. Peptides.

[37] Peñuela A., Robledo D., Bourgougnon N., Bedoux G., Hernández-Núñez E., Freile-Pelegrín Y. (2018). Environmentally Friendly Valorization of *Solieria filiformis* (Gigartinales, Rhodophyta) from IMTA Using a Biorefinery Concept. Mar. Drugs.

[38] Parera-Valadez Y., Yam-Puc A., López-Aguiar L.K., Borges-Argáez R., Figueroa-Saldivar M.A., Cáceres-Farfán M., Márquez-Velázquez N.A., Prieto-Davó A. (2019). Ecological Strategies Behind the Selection of Cultivable Actinomycete Strains from the Yucatan Peninsula for the Discovery of Secondary Metabolites with Antibiotic Activity. Microb. Ecol..

[39] Pless D.D., Aguilar M.B., Falcón A., Lozano-Alvarez E., Heimer de la Cotera E.P. (2003). Latent Phenoloxidase Activity and N-Terminal Amino Acid Sequence of Hemocyanin from *Bathynomus giganteus*, a Primitive Crustacean. Arch. Biochem. Biophys..

[40] Méndez Alpuche A.A. (2017). Caracterización Fisicoquímica de Biopolímeros Obtenidos del Exoesqueleto de la Cacerolita de Mar, *Limulus polyphemus*. Master’s Thesis.

[41] Rinehart K.L., Kishore V., Bible K.C., Sakai R., Sullins D.W., Li K. (1988). Didemnins and Tunichlorin: Novel Natural Products from the Marine Tunicate *Trididemnum solidum*. J. Nat. Prod..

[42] Cruz-Hernández E., Rodríguez-Morales S. (2016). Sea Anemones from Yucatan Peninsula as Source of Bioactive Compounds. Toxicol. Lett..

[43] Sánchez-Rodriguez J., Zugasti-Cruz A., Burnnet J.W. (2001). Cutaneous Stings from *Bartholomea annulata*. Contact Dermat..

[44] Santamaría A., Sánchez-Rodríguez J., Zugasti A., Martínez A., Galván-Arzate S., Segura-Puertas L. (2002). A Venom Extract from the Sea Anemone *Bartholomea annulata* Produces Haemolysis and Lipid Peroxidation in Mouse Erythrocytes. Toxicology.

[45] Sánchez-Rodríguez J., Zugasti A., Santamaría A., Galván-Arzate S., Segura-Puertas L. (2006). Isolation, Partial Purification and Characterization of Active Polypeptide from the Sea Anemone *Bartholomea annulata*. Basic Clin. Pharmacol. Toxicol..

[46] Morales-Landa J.L., Zapata-Pérez O., Cedillo-Rivera R., Segura-Puertas L., Simá-Alvarez R., Sánchez-Rodríguez J. (2007). Antimicrobial, Antiprotozoal, and Toxic Activities of Cnidarian Extracts from the Mexican Caribbean Sea. Pharm. Biol..

[47] Fenton-Navarro B., Arreguín L.B., García-Hernández E., Heimer E., Aguilar M.B., Rodríguez A.C., Arreguín-Espinosa R. (2003). Purification and Structural Characterization of Lectins from the Cnidarian *Bunodeopsis antillienis*. Toxicon.

[48] Monroy-Estrada H.I., Chirino Y.I., Soria-Mercado I.E., Sánchez-Rodríguez J. (2013). Toxins from the Caribbean Sea Anemone *Bunodeopsis globulifera* Increase Cisplatin-Induced Cytotoxicity of Lung Adenocarcinoma Cells. J. Venom. Anim. Toxins Trop. Dis..

[49] Flores-Pérez A.J., Sánchez-Rodríguez J. (2016). Isolation and Purification of Neurotoxins from the Caribbean Sea Anemone *Bunodeopsis globulifera*. Toxicol. Lett..

[50] Santos Y., Martínez M., Sandoval A., Rodríguez A.A., Falcón A., Heimer de la Cotera E.P., Aguilar M.B., Flores P., Felix R., Arreguín R. (2013). Arrhythmogenic Effect of a Crude Extract from Sea Anemone *Condylactis gigantea*: Possible Involvement of rErg1 Channels. Toxicon.

[51] Sánchez-Rodríguez J., Cruz-Vazquez K. (2006). Isolation and Biological Characterization of Neurotoxic Compounds from the Sea Anemone *Lebrunia danae* (Duchassaing and Michelotti, 1860). Arch. Toxicol..

[52] Monroy-Estrada H.I., Segura-Puertas L., Galván-Arzate S., Santamaría A., Sánchez-Rodríguez J. (2007). The Crude Venom from the Sea Anemone *Stichodactyla helianthus* Induces Haemolysis and Slight Peroxidative Damage in Rat and Human Erythrocytes. Toxicol. In Vitro.

[53] García-Arredondo A., Rojas-Molina A., Ibarra-Alvarado C., Iglesias-Prieto R. (2011). Effects of Bleaching on the Pharmacological and Toxicological Activities Elicited by the Aqueous Extracts Prepared from Two “Fire Corals” Collected in the Mexican Caribbean. J. Exp. Mar. Biol. Ecol..

[54] Hernández-Matehuala R., Vuelvas-Solórzano A.A., Zepeda-Rodríguez A., Palma L., Rojas A. (2012). Acute Toxicity and Brine Shrimp Cytotoxicity Induced by the Venom of the Fire Coral *M. alcicornis* Collected in the Mexican Caribbean. Toxicon.

[55] García-Arredondo A., Rojas A., Ibarra-Alvarado C., Iglesias-Prieto R. (2012). A Comparison of the Structural Characteristics of the Nematocysts of the “Fire Corals” *Millepora alcicornis* and *M. complanata*, and Their Hemolytic and Vasoconstrictor Effects. Toxicon.

[56] Hernández-Matehuala R., Rojas-Molina A., Vuelvas-Solórzano A.A., Garcia-Arredondo A., Ibarra Alvarado C., Olguín-López N., Aguilar M. (2015). Cytolytic and Systemic Toxic Effects Induced by the Aqueous Extract of the Fire Coral *Millepora alcicornis* Collected in the Mexican Caribbean and Detection of Two Types of Cytolisins. J. Venom. Anim. Toxins Trop. Dis..

[57] Olguín-López N., Hérnandez-Elizárraga V.H., Hernández-Matehuala R., Cruz-Hernández A., Guevara-González R., Caballero-Pérez J., Ibarra-Alvarado C., Rojas-Molina A. (2019). Impact of El Niño-Southern Oscillation 2015–2016 on the Soluble Proteomic Profile and Cytolytic Activity of *Millepora alcicornis* (“Fire Coral”) from the Mexican Caribbean. PeerJ.

[58] Rojas A., Torres M., Rojas J.I., Feregrino A., Heimer-de la Cotera E.P. (2002). Calcium-Dependent Smooth Muscle Excitatory Effect Elicited by the Venom of the Hydrocoral *Millepora complanata*. Toxicon.

[59] Ibarra-Alvarado C., García J.A., Aguilar M.B., Rojas A., Falcón A., Heimer de la Cotera E.P. (2007). Biochemical and Pharmacological Characterization of Toxins Obtained from the Fire Coral *Millepora complanata*. Comp. Biochem. Phys..

[60] García-Arredondo A., Rojas A., Ibarra-Alvarado C., Bah M. (2012). Systemic Toxicity of the “Fire Coral” *Millepora complanata*: Isolation of a Non-Protein Vasoconstrictor Fraction with Lethal Activity in Mice. Toxicon.

[61] García-Arredondo A., Rojas-Molina A., Bah M., Ibarra-Alvarado C., Gallegos-Corona M.A., García-Servín M. (2015). Systemic Toxic Effects Induced by the Aqueous Extract of the Fire Coral *Millepora complanata* and Partial Purification of Thermostable Neurotoxins with Lethal Effects in Mice. Comp. Biochem. Phys..

[62] Hernández-Elizárraga V.H., Olguín-López N., Hernández-Matehuala R., Ocharán-Mercado A., Cruz-Hernández A., Guevara-González R.G., Caballero-Pérez J., Ibarra-Alvarado C., Sánchez-Rodríguez J., Rojas-Molina A. (2019). Comparative Analysis of the Soluble Proteome and the Cytolytic Activity of Unbleached and Bleached *Millepora complanata* (“Fire Coral”) from the Mexican Caribbean. Mar. Drugs.

[63] García-Arredondo A., Rojas-Molina A., Ibarra-Alvarado C., Lazcano-Pérez F., Arreguín-Espinosa R., Sánchez-Rodríguez J. (2016). Composition and Biological Activities of the Aqueous Extracts of Three Scleractinian Corals from the Mexican Caribbean: *Pseudodiploria strigosa*, *Porites astreoides* and *Siderastrea siderea*. J. Venom. Anim. Toxins Trop. Dis..

[64] Segura-Puertas L., Avila-Soria G., Sánchez-Rodríguez J., Ramos-Aguilar M.E., Burnett J.W. (2002). Some Toxinological Aspects of *Aurelia aurita* (Linné) from the Mexican Caribbean. J. Venom. Anim. Toxins.

[65] Ponce D., López-Vera E., Aguilar M.B., Sánchez-Rodríguez J. (2013). Preliminary Results of the in Vivo and in Vitro Characterization of a Tentacle Venom Fraction from the Jellyfish *Aurelia aurita*. Toxins.

[66] Sánchez-Rodríguez J., Torrens E., Segura-Puertas L. (2006). Partial Purification and Characterization of a Novel Neurotoxin and Three Cytolysins from Box Jellyfish (*Carybdea marsupialis*) Nematocyst Venom. Arch. Toxicol..

[67] Lazcano-Pérez F., Arellano R.O., Garay E., Arreguín-Espinosa R., Sánchez-Rodríguez J. (2017). Electrophysiological Activity of a Neurotoxic Fraction from the Venom of Box Jellyfish *Carybdea marsupialis*. Comp. Biochem. Phys..

[68] Torres M., Aguilar M.B., Falcón A., Sánchez L., Radwan F.F.Y., Burnett J.W., Heimer-de la Cotera E.P., Arellano R.O. (2001). Electrophysiological and Hemolytic Activity Elicited by the Venom of the Jellyfish *Cassiopea xamachana*. Toxicon.

[69] Orduña-Novoa K., Segura-Puertas L., Sánchez-Rodríguez J., Meléndez A., Nava-Ruíz C., Rembao D., Santamaria A., Galván-Arzate S. (2003). Possible Antitumoral Effect of the Crude Venom of *Cassiopea xamachana* (Cnidaria: Scyphozoa) on Tumors of the Central Nervous System Induced by N-Ethyl-N-Nitrosourea (ENU) in Rats. Proc. West. Pharmacol. Soc..

[70] Sánchez-Rodríguez J., Lucio-Martínez N.L. (2011). Isolation and Prepurification of Active Compounds in Venom from *Pelagia noctiluca* (Scyphozoa: Pelagiidae) from the Caribbean Sea. Cienc. Mar..

[71] González Vásquez J.M., Mena Rejón G.J., Quijano L. (2013). Obtención de Saponinas Triterpénicas Potencialmente Citotóxicas a Partir de la Pared Corporal de *Holothuria Mexicana*. Foro en Ciencias Químicas y Bioquímicas.

[72] Pérez-Vega J.A., Olivera-Castillo L., Gómez-Ruiz J.A., Hernández-Ledesma B. (2013). Release of Multifunctional Peptides by Gastrointestinal Digestion of Sea Cucumber (*Isostichopus badionotus*). J. Funct. Foods.

[73] Pérez Espadas A.R. (2014). Evaluación de la Actividad Citotóxica y Componentes del Pepino de Mar *Isostichopus Badionotus* (Selenka, 1867) del Litoral de la Península de Yucatán, Mexico. Ph.D. Thesis.

[74] Pérez-Espadas A.R., Verde-Star M.J., Rivas-Morales C., Oranday-Cárdenas A., Morales-Rubio M.E., León-Deniz L.V., Canul-Canché J., Quijano L. (2014). In Vitro Cytotoxic Activity of *Isostichopus badionotus*, A Sea Cucumber from Yucatan Peninsula Coast. J. Pharm. Nutr. Sci..

[75] Rojas A., Feregrino A., Ibarra-Alvarado C., Aguilar M.B., Falcon A., Heimer de la Cotera E.P. (2008). Pharmacological Characterization of Venoms Obtained from Mexican Toxoglossate Gastropods on Isolated Guinea Pig Ileum. J. Venom. Anim. Toxins Trop. Dis..

[76] Aguilar M.B., Pérez-Reyes L.I., López Z., Heimer de la Cotera E.P., Falcón A., Ayala C., Galván M., Salvador C., Escobar L.I. (2010). Peptide sr11a from *Conus spurius* is a Novel Peptide Blocker for Kv1 Potassium Channels. Peptides.

[77] López-Vera E., Heimer De La Cotera E.P., Maillo M., Riesgo-Escovar J.R., Olivera B.M., Aguilar M.B. (2004). A Novel Structural Class of Toxins: The Methionine-Rich Peptides from the Venoms of Turrid Marine Snails (Mollusca, Conoidea). Toxicon.

[78] Tello Cetina J., Chan Pat A., Rivera Muñoz G., Tamayo Cortes J., Jimenez Suaste N., Loria Sunsa H. (2018). Uso de la Melanina del Pulpo (*Octopus maya*) de Yucatán como Agente Antibacteriano. Revista Cubana Investigaciones Pesqueras.

[79] Zubia M., Robledo D., Freile-Pelegrin Y. (2007). Antioxidant Activities in Tropical Marine Macroalgae from the Yucatan Peninsula, Mexico. J. Appl. Phycol..

[80] Freile-Pelegrin Y., Robledo D., Chan-Bacab M.J., Ortega-Morales B.O. (2008). Antileishmanial Properties of Tropical Marine Algae Extracts. Fitoterapia.

[81] Moo-Puc R., Robledo D., Freile-Pelegrin Y. (2008). Evaluation of Selected Tropical Seaweeds for in Vitro Anti-Trichomonal Activity. J. Ethnopharmacol..

[82] Moo-Puc R., Robledo D., Freile-Pelegrín Y. (2009). Actividad Citotóxica y Antiproliferativa in Vitro de Macroalgas Marinas de Yucatán, Mexico. Cienc. Mar..

[83] León-Deniz L.V., Dumonteil E., Moo-Puc R., Freile-Pelegrin Y.A. (2009). Antitrypanosomal in Vitro Activity of Tropical Marine Algae Extracts. Pharm. Biol..

[84] Morales J.L., Cantillo-Ciau Z.O., Sánchez-Molina I., Mena-Rejón G.J. (2006). Screening of Antibacterial and Antifungal Activities of Six Marine Macroalgae from Coasts of Yucatán Peninsula. Pharm. Biol..

[85] Freile-Pelegrin Y., Morales J.L. (2004). Antibacterial Activity in Marine Algae from the Coast of Yucatan, Mexico. Bot. Mar..

[86] De Lara-Isassi G., Álvarez-Hernández S., Collado-Vides L. (2000). Ichtyotoxic Activity of Extracts from Mexican Marine Macroalgae. J. Appl. Phycol..

[87] García Granados R.U. (2015). Efecto Hipoglucémico, Hipolipidémico y Citotóxico de Macroalgas y Pastos Marinos del Golfo de México. Master’s Thesis.

[88] Robledo D., Freile Pelegrín Y. (1997). Chemical and Mineral Composition of Six Potentially Edible Seaweed Species of Yucatan. Bot. Mar..

[89] Gomez Hernandez M. (2018). Actividad Antifúngica de Extractos de Macroalgas Marinas de La Costa de Yucatán. Master’s Thesis.

[90] Moo-Puc R., Robledo D., Freile-Pelegrin Y. (2011). Improved Antitumoral Activity of Extracts Derived from Cultured *Penicillus dumetosus*. Trop. J. Pharm. Res..

[91] Moo-Puc R., Robledo D., Freile-Pelegrin Y. (2011). Enhanced Antitumoral Activity of Extracts Derived from Cultured *Udotea flabellum* (Chlorophyta). Evid.-Based Complement. Altern. Med..

[92] García-Ríos V., Ríos-Leal E., Robledo D., Freile-Pelegrin Y. (2012). Polysaccharides Composition from Tropical Brown Seaweeds. Phycol. Res..

[93] Caamal-Fuentes E., Chale-Dzul J., Moo-Puc R., Freile-Pelegrin Y., Robledo D. (2014). Bioprospecting of Brown Seaweed (Ochrophyta) from the Yucatan Peninsula: Cytotoxic, Antiproliferative, and Antiprotozoal Activities. J. Appl. Phycol..

[94] Chale-Dzul J., Freile-Pelegrín Y., Robledo D., Moo-Puc R. (2017). Protective Effect of Fucoidans from Tropical Seaweeds against Oxidative Stress in HepG2 Cells. J. Appl. Phycol..

[95] Bedoux G., Caamal-Fuentes E., Boulho R., Marty C., Bourgougnon N., Freile-Pelegrín Y., Robledo D. (2017). Antiviral and Cytotoxic Activities of Polysaccharides Extracted from Four Tropical Seaweed Species. Nat. Prod. Commun..

[96] Quintal-Novelo C., Rangel-Méndez J., Ortiz-Tello Á., Graniel-Sabido M., Pérez-Cabeza de Vaca R., Moo-Puc R. (2018). A *Sargassum Fluitans* Borgesen Ethanol Extract Exhibits a Hepatoprotective Effect in Vivo in Acute and Chronic Liver Damage Models. BioMed Res. Int..

[97] Chale-Dzul J., Moo-Puc R., Robledo D., Freile-Pelegrín Y. (2014). Hepatoprotective Effect of the Fucoidan from the Brown Seaweed *Turbinaria tricostata*. J. Appl. Phycol..

[98] Freile-Pelegrín Y., Robledo D., Azamar J.A. (2006). Carrageenan of *Eucheuma isiforme* (Solieriaceae, Rhodophyta) from Yucatán, Mexico. I. Effect of Extraction Conditions. Bot. Mar..

[99] Freile-Pelegrín Y., Murano E. (2005). Agars from Three Species of *Gracilaria* (Rhodophyta) from Yucatán Peninsula. Bioresour. Technol..

[100] Espinoza-Avalos J., Hernández-Garibay E., Zertuche-González J.A., Meave del Castillo M.E. (2003). Agar de Dos Especies Coexistentes de *Gracilaria* (Gracilariaceae) del Caribe Mexicano. Cienc. Mar..

[101] Zubia M., Freile-Pelegrín Y., Robledo D. (2014). Photosynthesis, Pigment Composition and Antioxidant Defences in the Red Alga *Gracilariopsis tenuifrons* (Gracilariales, Rhodophyta) under Environmental Stress. J. Appl. Phycol..

[102] Freile-Pelegrín Y., Azamar J.A., Robledo D. (2011). Preliminary Characterization of Carrageenan from the Red Seaweed *Halymenia floresii*. J. Aquat. Food Prod. Technol..

[103] Godínez Ortega J.L., Robledo Ramírez D., Freile Pelegrín Y., Ríos Castillo T. (2012). Seasonal Fatty Acid Composition of *Halymenia floresii* (Rhodophyta) in Yucatan, Mexico. Revista Latinoamericana Quimica.

[104] Vázquez-Delfín E., Robledo D., Freile-Pelegrín Y. (2014). Microwave-assisted Extraction of the Carrageenan from *Hypnea musciformis* (Cystocloniaceae, Rhodophyta). J. Appl. Phycol..

[105] Peralta-García E., Caamal-Fuentes E., Robledo D., Hernández-Núñez E., Freile-Pelegrín Y. (2016). Lipid Characterization of Red Alga *Rhodymenia pseudopalmata* (Rhodymeniales, Rhodophyta). Phycol. Res..

[106] Pliego-Cortés H., Caamal-Fuentes E., Montero-Muñoz J., Freile-Pelegrín Y., Robledo D. (2017). Growth, Biochemical and Antioxidant Content of *Rhodymenia pseudopalmata* (Rhodymeniales, Rhodophyta) Cultivated under Salinity and Irradiance Treatments. J. Appl. Phycol..

[107] Morán-Santibañez K., Cruz-Suárez L.E., Ricque-Marie D., Robledo D., Freile-Pelegrín Y., Peña-Hernández M.A., Rodríguez-Padilla C., Trejo-Avila L.M. (2016). Synergistic Effects of Sulfated Polysaccharides from Mexican Seaweeds against Measles Virus. Biomed. Res. Int..

[108] Caamal-Fuentes E., Robledo D., Freile-Pelegrín Y. (2017). Physicochemical Characterization and Biological Activities of Sulfated Polysaccharides from Cultivated *Solieria filiformis* Rhodophyta. Nat. Prod. Commun..

[109] WoRMS Editorial Board (2019). World Register of Marine Species. http://www.marinespecies.org.

